# EZH2 Inhibition to Counteract Oral Cancer Progression through Wnt/β-Catenin Pathway Modulation

**DOI:** 10.3390/ph17081102

**Published:** 2024-08-22

**Authors:** Michela Campolo, Sarah Adriana Scuderi, Alessia Filippone, Valentina Bova, Sofia Paola Lombardo, Lorenzo Colarossi, Serena Sava, Anna Paola Capra, Federica De Gaetano, Marco Portelli, Angela Militi, Emanuela Esposito, Irene Paterniti

**Affiliations:** 1 Department of Chemical, Biological, Pharmaceutical and Environmental Sciences, University of Messina, Viale Ferdinando Stagno D’Alcontres, 31, 98166 Messina, ME, Italy; michela.campolo@unime.it (M.C.); sascuderi@unime.it (S.A.S.); alessia.filippone@unime.it (A.F.); valentina.bova@unime.it (V.B.); annapaola.capra@unime.it (A.P.C.); fedegaetano@unime.it (F.D.G.); ipaterniti@unime.it (I.P.); 2 Istituto Oncologico del Mediterraneo, Via Penninazzo 7, 95029 Viagrande, CT, Italy; sofia.lombardo@grupposamed.com (S.P.L.);lorenzo.colarossi@grupposamed.com (L.C.); serena.sava@grupposamed.com (S.S.); 3 Department of Biomedical and Dental Science, Morphological and Functional Images, University of Messina, Via Consolare Valeria, 98125 Messina, ME, Italy; marco.portelli@unime.it (M.P.); angela.militi@unime.it (A.M.)

**Keywords:** oral squamous cell carcinoma, enhancer of zeste homolog 2, inflammation, angiogenesis, Wnt/β-catenin signaling pathway

## Abstract

Oral squamous cell carcinoma (OSCC) is one of the most common human malignancies worldwide. The molecular mechanisms of OSCC pathogenesis are still unknown; however, in recent years, several reports have focused on the role of enhancer of zeste homolog 2 (EZH2) in OSCC. Therefore, in this study we aimed to investigate the effects of GSK343, a selective EZH2 inhibitor, and its impact on the signaling pathways in OSCC, using an in vitro and in vivo orthotopic model. In the in vitro model, GSK343 (1, 10, and 25 μM) significantly decreased OSCC cell viability and cell migration through EZH2 inhibition, modulating NF-κB/IκBα pathway activation and eNOS, VEGF, and TGFβ expression, important markers of angiogenesis. In the in vivo model, GSK343 (5 mg/kg and 10 mg/kg) restored tongue tissue architecture and reduced tumor progression through EZH2 inhibition and Wnt/β-catenin signaling pathway modulation. Moreover, GSK343 reduced the expression of inflammatory mediators; eNOS and TGFβ, markers of angiogenesis; and CD31 and CD34, markers of micro vessel density, respectively. In conclusion, our data demonstrate that GSK343 counteracts oral cancer progression through EZH2/Wnt/β-catenin pathway modulation, suggesting that it could be a promising therapeutic approach for OSCC management.

## 1. Introduction

About 890,000 new cases of head and neck squamous cell carcinoma (HNSCC) are reported annually, making it the sixth most common malignancy worldwide [1,2]. HNSCC includes several tumors originating from the squamous epithelium of the oral cavity, oropharynx, larynx, and hypopharynx [3]. Oral squamous cell carcinoma (OSCC) is an aggressive HNSCC tumor that is characterized by recurrence and lymph node metastasis (LNM) [3]. OSCC treatment involves surgical resection, radiotherapy, and chemotherapy [4]. Despite the numerous developments that have been made in its diagnosis and management, the survival rate of patients with OSCC is still disappointing [5]. Therefore, it is of crucial importance to identify novel therapeutic targets and to find diagnostic strategies with which to improve OSCC treatment [5]. Enhancer of zeste homolog 2 (EZH2), an essential catalytic subunit of the polycomb 2 repressive complex (PRC2), has been reported to be associated with OSCC carcinogenesis and adverse outcomes in patients with oral cancer [6,7]. EZH2 is a methyltransferase that acts on histone H3 lysine27 (H3K27) to produce trimethylated H3K27 (H3K27me3), which consequently regulates chromatin compaction and gene inactivation [8,9]. In the context of oral cancer, some studies have made a significant contribution to the understanding of EZH2’s role in OSCC [6,7]. In particular, Zhao and colleagues [6] measured the levels of EZH2 in several OSCC cell lines and tissue specimens, demonstrating the presence of higher EZH2 levels in them than in non-cancerous cells. Specifically, it has been proven that knocking down EZH2 reduces OSCC cell proliferation and induces apoptosis, proving its involvement in cancer progression [6]. Another study by Cao et al. [7] demonstrated the possible use of EZH2 as a biomarker for predicting OSCC development in patients with oral leukoplakia.

EZH2 is well-known to be a regulator of autophagy, apoptosis, and cell proliferation, as it is involved in numerous signaling pathways, including the Wnt/β-catenin pathway [10,11]. Specifically, the canonical Wnt/β-catenin pathway, belonging to the Wnt pathways, is activated via the binding of extracellular Wnt ligands to a receptor complex comprising the Frizzled (Fz) and low-density lipoprotein receptor-related protein 5/6 (LRP5/6) [12]. Once activated, the canonical Wnt pathway induces the nuclear translocation of β-catenin and the activation of numerous target genes involved in several biological processes, such as cell proliferation, survival, and migration [12]. Among these processes, Liu and colleagues [13] have proven that the Wnt/β-catenin pathway may stimulate the phosphorylation of signal transducer and activator of transcription 3 (STAT3), which has been shown to be associated with tumor growth in and a poor prognosis for OSCC patients [11,14].

EZH2 also controls the transcription of several inflammatory genes that promote carcinogenesis, including nuclear factor κ-light-chain-enhancer of activated B cells (NF-κB) [15]. As has been profusely discussed, inflammation plays a key role in different phases of tumor development: initiation, promotion, invasion, and metastasis [16]. Various studies have shown high levels of pro-inflammatory cytokines, such as tumor necrosis factor-α (TNF-α), in oral cancer patients and in the saliva of patients with pre-neoplastic oral lesions; however, the exact mechanisms by which inflammation regulates oral cancer progression through EZH2 are still unknown [16,17,18]. 

Considering the role of EZH2 in cancer, numerous EZH2 inhibitors have been created, such as 3-deazaneplanocin A (DZNep) and GlaxoSmithKline 343 (GSK343) [10]. GSK343 is a competitive methionine histone lysine methyl-transferase inhibitor that selectively inhibits EZH2 activity or expression [19]. Various reports have demonstrated that GSK343 counteracts the progression of various cancer types, including epithelial ovarian cancer, pancreatic cancer, and oral cancer, by modulating the p16/p21 pathway and epithelial–mesenchymal transition (EMT) [19,20,21,22].

However, despite these reports, additional studies are needed to better understand its effect in the context of OSCC.

Recently, we evaluated the effect of GSK343 on a model of glioblastoma, showing how the compound was able to reduce tumor progression via the NF-κB/IκBα pathway [23]. 

Thus, based on these findings, the purpose of this study was to investigate the effects of GSK343 on oral cancer and to study in depth its molecular mechanism, focusing on the Wnt/β-catenin pathway to obtain a broader knowledge about its effects, using an in vitro and in vivo model.

## 2. Results

### 2.1. GSK343 Treatment Reduces OSCC Cell Viability

**Figure 1:**
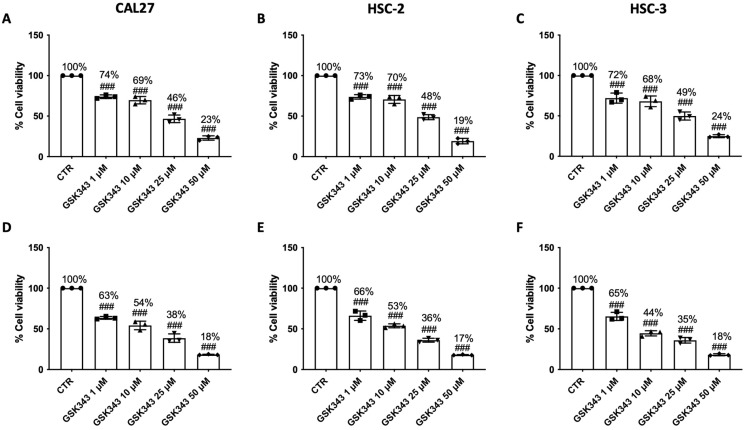
GSK343′s impact on OSCC cell viability. MTT assay was performed to evaluate the cytotoxicity of GSK343 (1, 10, 25, and 50 μM) after 24 h (**A**–**C**) and 48 h (**D**–**F**) of treatment in CAL27, HSC-2, and HSC-3 cells. The data were obtained from three separate experiments. (**A**–**F**) ###: *p* < 0.001 vs. control group.

Cell viability assay demonstrated that treatment with GSK343 for 24 h and 48 h (1, 10, and 25 μM) was able to decrease OSCC cell viability, exerting a marked cytotoxic effect on CAL27, HSC-2, and HSC-3 cell lines at 24 h (Figure 1A–C), and especially at 48 h (Figure 1D–F). GSK343, at a concentration of 50 μM, demonstrated to reduce OSCC cell viability more than 24% at 24 h and 18% at 48 h; thus, because of this, we decided to just assess the effects of GSK343 only at lower concentrations (1, 10, and 25 μM) at 24 h to reduce the overall level of toxicity, and only on CAL27 cells, one of the most utilized cell lines in the context of oral cancer [24,25,26]. 

GSK343 did not exert any cytotoxic effect on non-cancerous HOK cells at 24 h; however, the highest concentration of GSK343 (50 μM) reduced HOK cell viability to 82% (Supplementary Figure S1A). After 48 h of treatment with GSK343 at concentrations of 1, 10, and 25 μM, the HOK cells showed decreased cell viability, from 84% to 71%; however, the highest concentration (50 μM) reduced HOK cell viability to 69% (Supplementary Figure S1B).

The values of LC50 of GSK343 in CAL27, HSC-2, and HSC-3 cells at 24 h were 1.13, 1.09, and 1.19 μM, respectively.

### 2.2. GSK343 Treatment Reduces CAL27 Cells Migration 

**Figure 2:**
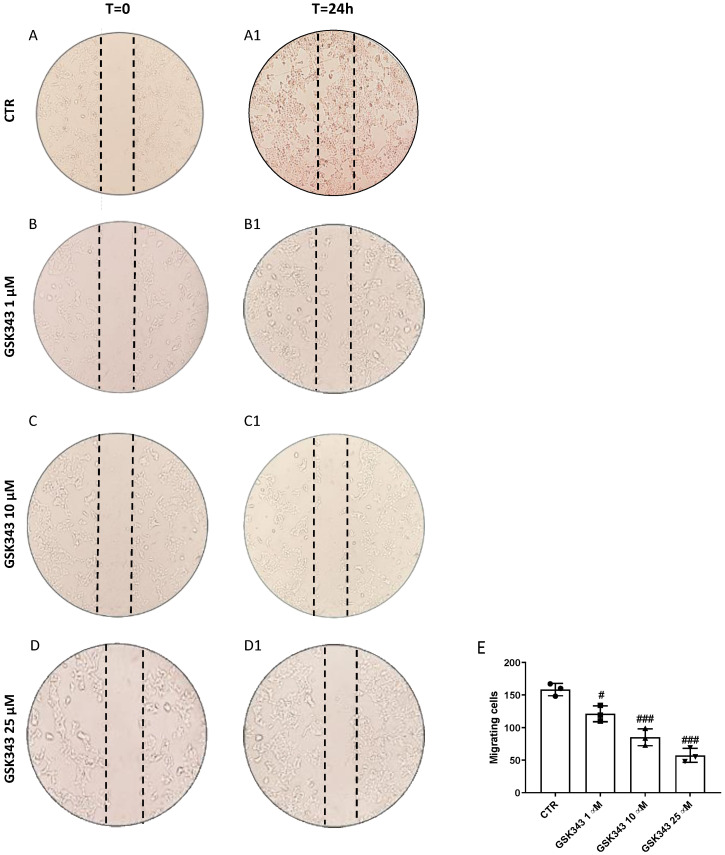
Evaluation of GSK343 on CAL27 cells migration. Scratch assay was performed in CAL27 cells at T = 0 (**A**–**D**). GSK343 treatment for 24 h (1, 10, and 25 μM) significantly decreased CAL27 cells migration (**B1**–**D1**) compared to that of control group ((**A1**); migrating cells score (**E**)). Data were obtained from three separate experiments. (**E**) #: *p* < 0.05 vs. control group; ###: *p* < 0.001 vs. control group.

Scratch assay was performed to evaluate the ability of GSK343 to reduce CAL27 cells migration. GSK343 (1, 10, and 25 μM) decreased cell migration into the scratched area in a concentration-dependent manner (Figure 2(B1–D1)) compared to a control group at T = 24 h (Figure 2(A1); see the migrating cells score in Figure 2E) and compared to the T = 0 time point (Figure 2A–D).

### 2.3. GSK343 Treatment Reduces EZH2, H3K27me3, and Nf-κB/IκBα Pathway Activation in CAL27 Cells

**Figure 3:**
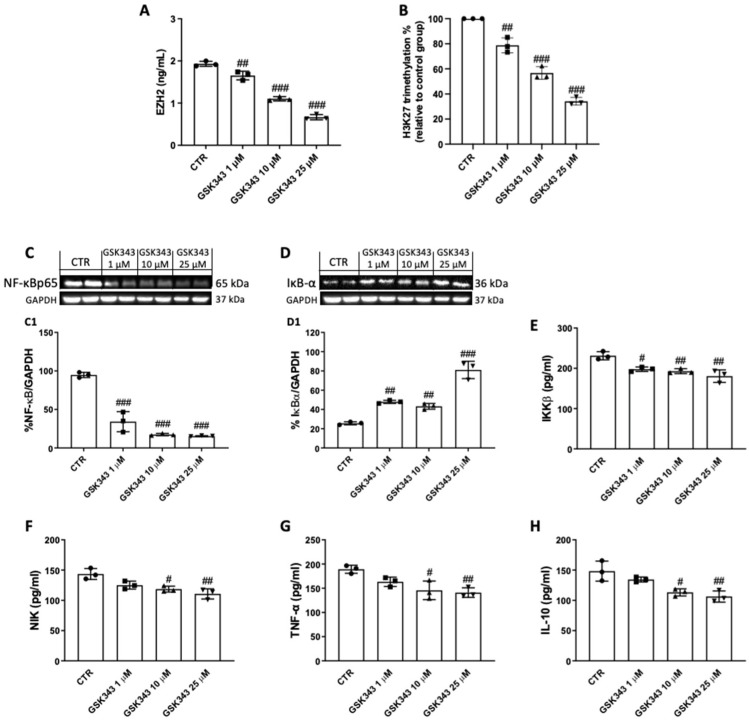
GSK343′s impact on *EZH2*, *H3K27me3*, and *NF-κB/IκBα* pathway in CAL27 cells. ELISA kits were used and western blot analyses were performed to assess the impact of GSK343 on EZH2 (**A**), H3K27me3 (**B**), NF-κB (**C**,**C1**), IκB-α (**D**,**D1**), IKKβ (**E**), NIK (**F**), TNF-α (**G**), and IL-10 (**H**) levels in CAL27 cells. Data were obtained from three separate experiments. (**A**) ##: *p* < 0.01 vs. control group; ###: *p* < 0.001 vs. control group. (**B**) ##: *p* < 0.01 vs. control group; ###: *p* < 0.001 vs. control group. (**C**) ###: *p* < 0.001 vs. control group. (**D**) ##: *p* < 0.01 vs. control group; ###: *p* < 0.001 vs. control group. (**E**) #: *p* < 0.05 vs. control group; ##: *p* < 0.01 vs. control group. (**F**) #: *p* < 0.05 vs. control group; ##: *p* < 0.01 vs. control group; (**G**) #: *p* < 0.05 vs. control group; ##: *p* < 0.01 vs. control group. (**H**) #: *p* < 0.05 vs. control group; ## *p* < 0.01 vs. control group.

GSK343 demonstrated to significantly reduce EZH2 levels (Figure 3A), as well as the percentage of H3K27 trimethylation (Figure 3B), in CAL27 cells after 24 h of treatment. 

Moreover, treatment with GSK343 for 24 h (1, 10, and 25 μM) significantly decreased NF-κB (Figure 3C(C1)) and reestablished IκB-α expression (Figure 3D(D1)) compared to the control group, and also decreased IKKβ (Figure 3E) and NIK levels (Figure 3F). In addition, GSK343 (10 and 25 μM) reduced the levels of inflammatory cytokines TNF-α (Figure 3G) and IL-10 (Figure 3H).

### 2.4. GSK343 Treatment Reduces NO_x_, iNOS, eNOS, VEGF, and TGFβ Levels in CAL27 Cells

**Figure 4:**
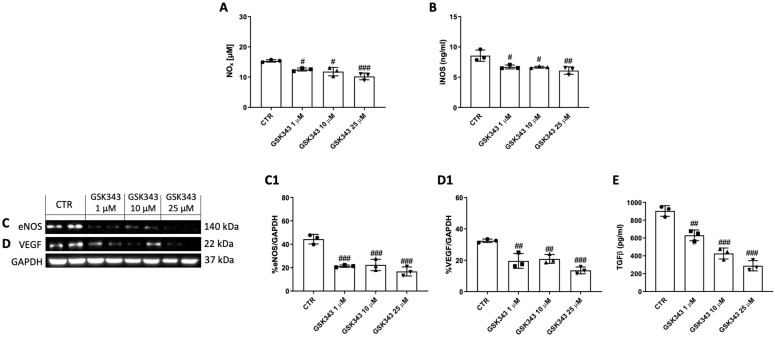
GSK343′s impact on NO_x_, iNOS, eNOS, VEGF, and TGFβ levels in CAL27 cells. GSK343 treatment (1, 10, and 25 μM) significantly reduced NO_x_ (**A**) and iNOS levels (**B**) compared to control group. Moreover, GSK343, at same concentrations, reduced eNOS (**C**,**C1**) and VEGF (**D**,**D1**) expression, and TGFβ (**E**) levels. Data were obtained from three separate experiments. (**A**) #: *p* < 0.05 vs. control group; ###: *p* < 0.001 vs. control group. (**B**) #: *p* < 0.05 vs. control group; ##: *p* < 0.01 vs. control group. (**C**) ###: *p* < 0.001 vs. control group. (**D**) ##: *p* < 0.01 vs. control group; ###: *p* < 0.001 vs. control group. (**E**) ##: *p* < 0.01 vs. control group; ###: *p* < 0.001 vs. control group.

Treatment with GSK343 for 24 h (1, 10, and 25 μM) significantly reduced NO_x_ and iNOS levels compared to the control group (Figure 4A,B). Moreover, GSK343 treatment significantly decreased the expression of eNOS and VEGF, and the levels of TGFβ compared to the control group, as shown in Figure 4C(C1),D(D1),E.

### 2.5. GSK343 Treatment Increases Apoptosis Process in CAL27 Cells

**Figure 5:**
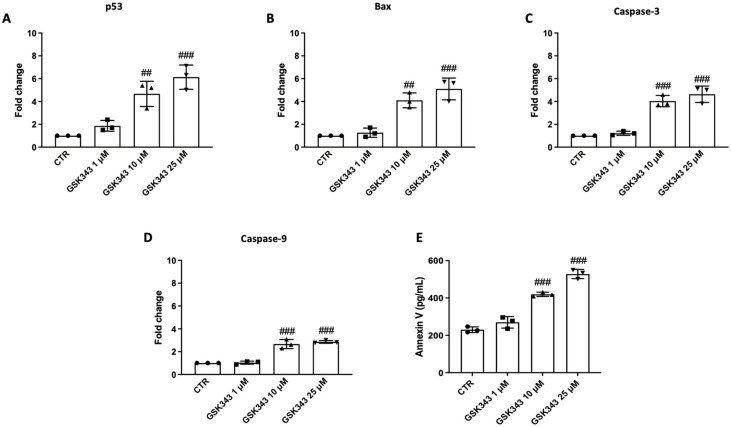
GSK343′s impact on p53, Bax, caspase-3, caspase-9, and Annexin V levels in CAL27 cells. GSK343 increased p53, Bax, caspase-3, and caspase-9 levels (**A**–**D**). Annexin V ELISA kit was used to confirm the effect of GSK343 on apoptosis after 24 h of treatment (**E**). Data were obtained from three separate experiments. (**A**) ##: *p* < 0.01 vs. control group; ###: *p* < 0.001 vs. control group. (**B**) ##: *p* < 0.01 vs. control group; ###: *p* < 0.001 vs. control group. (**C**) ###: *p* < 0.001 vs. control group. (**D**) ###: *p* < 0.001 vs. control group. (**E**) ###: *p* < 0.001 vs. control group.

RT-qPCR was used to evaluate GSK343′s impact on apoptosis. The results demonstrate that treatment with GSK343 for 24 h (10 and 25 μM) augmented the pro-apoptotic levels of p53, Bax, caspase-3, and caspase-9 (Figure 5A–D). Moreover, GSK343 significantly increased Annexin V levels compared to the control group (Figure 5E).

### 2.6. GSK343 Treatment Reduces In Vivo Tumor Growth through EZH2 Inhibition 

**Figure 6:**
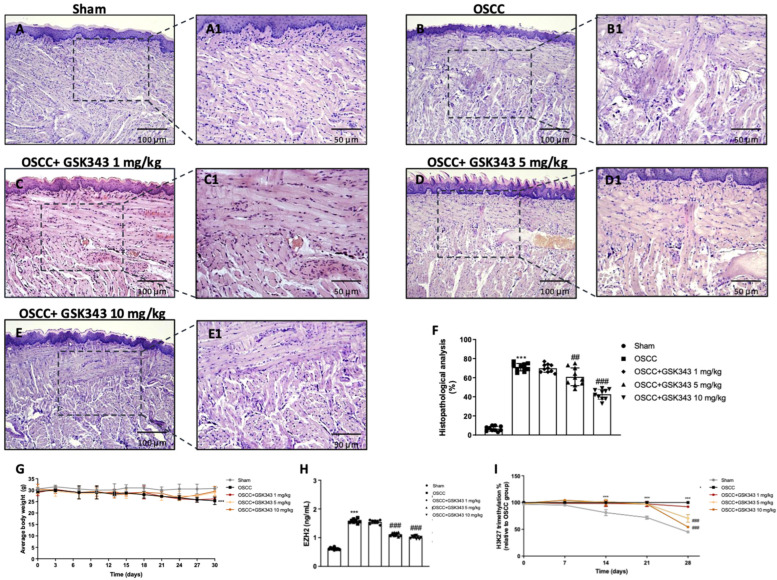
GSK343′s impact on tumor growth using an OSCC orthotopic model. The OSCC group showed the presence of larger and more pronounced cell nuclei and dysplasia in the tongue tissue (**B**,**B1**) compared to the sham group (**A**,**A1**). Conversely, GSK343 (5 and 10 mg/kg) significantly reduced these (**D**,**D1**,**E**,**E1**) more than treatment with 1 mg/kg ((**C**,**C1**); see histopathological analysis score (**F**)), reestablishing the animals’ body weight (**G**). GSK343 (5 and 10 mg/kg) significantly reduced EZH2 levels (**H**) and the percentage of H3K27 trimethylation (**I**). (**F**) ***: *p* < 0.001 vs. sham; ##: *p* < 0.01 vs. OSCC group; ###: *p* < 0.001 vs. OSCC group. (**G**) ***: *p* < 0.001 vs. sham. (**H**) ***: *p* < 0.001 vs. sham; ###: *p* < 0.001 vs. OSCC group. (**I**) ***: *p* < 0.001 vs. sham; ###: *p* < 0.001 vs. OSCC group.

Histological evaluation demonstrated the presence of larger and more pronounced cell nuclei, their irregular size, and dysplasia in the tongues of the OSCC group (Figure 6B(B1)) compared to those of the sham group (Figure 6A(A1)). Nonetheless, treatment with GSK343 (5 mg/kg and 10 mg/kg) decreased these in comparison with those of the OSCC group (Figure 6D,E(D1,E1)). Treatment with 1 mg/kg did not exert any positive effect (Figure 6C (C1); see histopathological score in Figure 6F). Furthermore, the OSCC group showed a decrease in body weight; however, GSK343 (5 mg/kg and 10 mg/kg) reestablished it, but not significantly (Figure 6G). GSK343 (5 mg/kg and 10 mg/kg) decreased EZH2 levels in the tongue homogenates compared to the OSCC group (Figure 6H), as well as the percentage of H3K27 trimethylation in the serum samples from day 21, as shown in Figure 6I. The percentage of mice survival was 100%.

### 2.7. GSK343 Treatment Reduces In Vivo Wnt/β-Catenin Pathway Activation 

**Figure 7:**
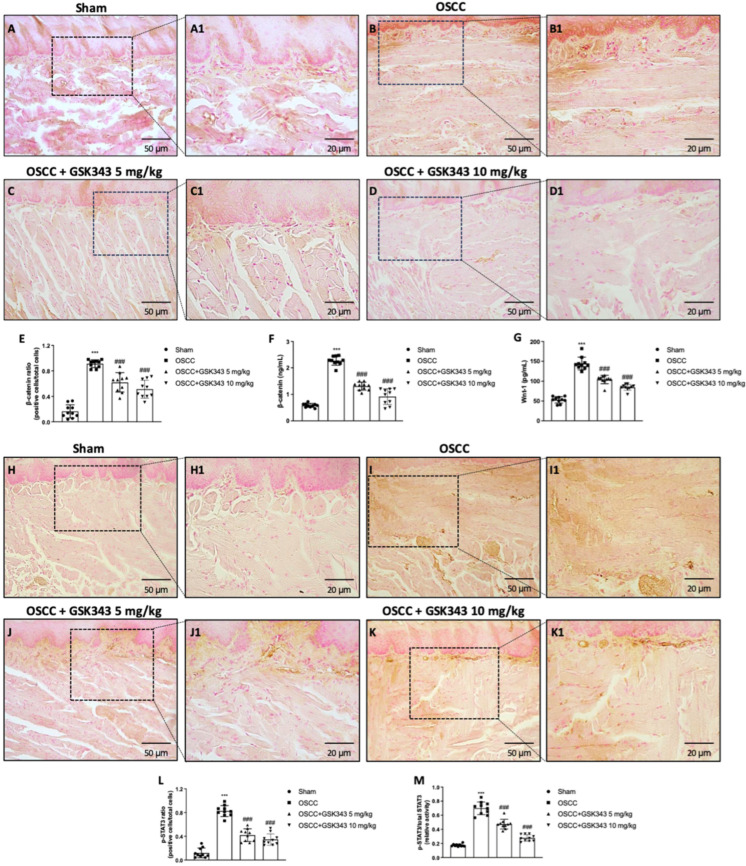
GSK343′s impact on Wnt/β-catenin pathway in vivo. GSK343 significantly decreased number of β-catenin-positive cells (**C**,**C1**,**D**,**D1**) compared to OSCC group ((**B**,**B1**) and sham group (**A**,**A1**); see immunohistochemical score in (**E**)). Data from immunohistochemistry were confirmed by ELISA kit (**F**). GSK343 significantly reduced Wnt-1 levels (**G**) and p-STAT3-positive cells (**J**,**J1**,**K**,**K1**) compared to OSCC group ((**I**,**I1**) and sham group (**H**,**H1**); see immunohistochemical score in (**L**)). Results for p-STAT3 were confirmed by ELISA kit (**M**). (**E**) ***: *p* < 0.001 vs. sham; ###: *p* < 0.001 vs. OSCC group. (**F**) ***: *p* < 0.001 vs. sham; ###: *p* < 0.001 vs. OSCC group. (**G**) ***: *p* < 0.001 vs. sham; ###: *p* < 0.001 vs. OSCC group. (**L**) ***: *p* < 0.001 vs. sham; ###: *p* < 0.001 vs. OSCC group. (**M**) ***: *p* < 0.001 vs. sham; ###: *p* < 0.001 vs. OSCC group.

Based on the role of EZH2 in Wnt/β-catenin signaling pathway, we decided to evaluate the effect of GSK343 on β-catenin and Wnt-1 by an immunohistochemical analysis and ELISA kit, respectively. GSK343 at both doses diminished the number of β-catenin-positive cells (Figure 7C,D(C1,D1)) compared to the OSCC group (Figure 7B(B1); see immunohistochemical score in Figure 7E). The data were confirmed by an ELISA kit as shown in Figure 7F. Additionally, GSK343 was able to reduce Wnt-1 levels compared to the untreated OSCC group (Figure 7G). 

Moreover, we evaluated p-STAT3 by immunohistochemistry, and showed that GSK343 significantly reduced the number of p-STAT3-positive cells (Figure 7J,K(J1,K1)) compared to the OSCC group (Figure 7I(I1); see the immunohistochemical score in Figure 7L). These results were confirmed by an ELISA kit, as shown in Figure 7M.

### 2.8. GSK343 Treatment Decreases In Vivo NF-κB/IκBα Pathway Activation

**Figure 8:**
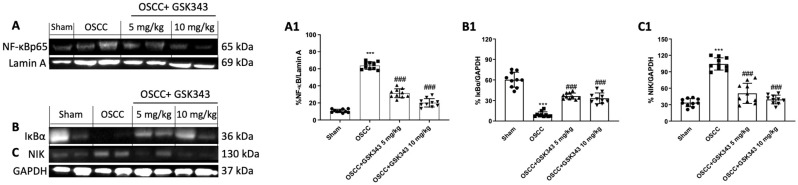
GSK343′s impact on inflammatory pathway in vivo. GSK343 (5 and 10 mg/kg) significantly decreased NF-κB/IκB-α pathway activation (**A**,**A1**,**B**,**B1**) compared to OSCC group. In addition, GSK343 at both doses diminished NIK expression (**C**,**C1**). (**A**) ***: *p* < 0.001 vs. sham; ###: *p* < 0.001 vs. OSCC group. (**B**) ***: *p* < 0.001 vs. sham; ###: *p* < 0.001 vs. OSCC group. (**C**) ***: *p* < 0.001 vs. sham; ###: *p* < 0.001 vs. OSCC group.

We chose to assess the impact of GSK343 on the NF-κB/IκBα pathway by an OSCC orthotopic model. Our data demonstrate that GSK343 treatment at both doses decreased NF-κB nuclear translocation and reestablished IκBα expression in the cytoplasm compared to the OSCC group, as shown in Figure 8A,B(A1,B1). Furthermore, the western blot analysis demonstrated an increase in NIK expression, an important kinase involved in non-canonical NF-κB pathway activation [27], in the OSCC group compared to the sham group; however, GSK343 treatment reduced its expression (Figure 8C(C1)).

### 2.9. GSK343 Treatment Reduces In Vivo eNOS, TGFβ, CD31, and CD34 Expression 

**Figure 9:**
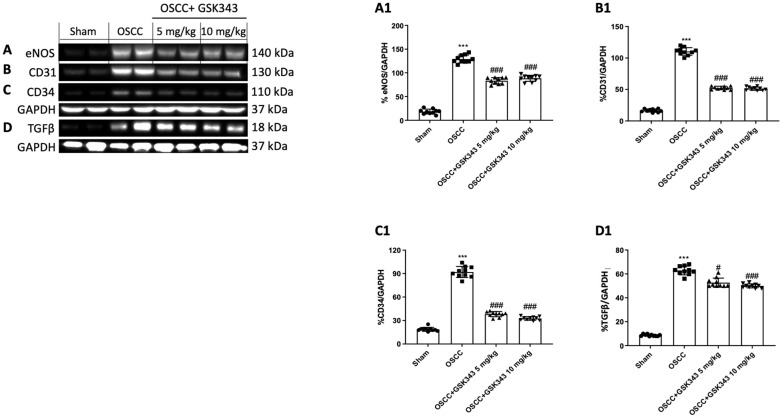
GSK343′s impact on eNOS, TGFβ, CD31, and CD34 expression in vivo. Immunoblotting was carried out to evaluate the impact of GSK343 on eNOS (**A**,**A1**), CD31 (**B**,**B1**), CD34 (**C**,**C1**), and TGFβ (**D**,**D1**) expression in tongue tissue. (**A**) ***: *p* < 0.001 vs. sham; ###: *p* < 0.001 vs. OSCC group. (**B**) ***: *p* < 0.001 vs. sham; ###: *p* < 0.001 vs. OSCC group. (**C**) ***: *p* < 0.001 vs. sham; ###: *p* < 0.001 vs. OSCC group. (**D**) ***: *p* < 0.001 vs. sham; #: *p* < 0.05 vs. OSCC group; ###: *p* < 0.001 vs. OSCC group.

Considering the critical role that EZH2 plays in tumor angiogenesis [28], we examined the effect of GSK343 on eNOS and TGFβ expression by western blot analysis. Our data demonstrate that the OSCC group was associated with a higher expression of eNOS and TGFβ in comparison with the sham group; nonetheless, GSK343 at both doses markedly decreased their expression (Figure 9A,D(A1,D1)).

Moreover, we decided to evaluate the effects of GSK343 on CD31 and CD34 expression, two important markers of micro vessel density [29,30,31,32,33], by western blot analysis. The OSCC group demonstrated a greater expression of CD31 and CD34 than the sham group did; nonetheless, GSK343 treatment at both doses significantly decreased their expression (Figure 9B,C(B1,C1)).

## 3. Discussion

The discovery of new molecular targets represents an important goal for the management of OSCC, whose incidence rate is continuously increasing [34]. It has been revealed that EZH2 plays a pivotal role in OSCC pathogenesis [6,7,14]. In particular, studies by Zhao [6] and Cao [7] have contributed greatly to the knowledge of EZH2’s function in oral cancer, suggesting its possible use as a biomarker as well. Although numerous molecular mechanisms of EZH2 have been elucidated, not enough is known about the crosstalk between EZH2 and the Wnt/β-catenin signaling pathway in OSCC. Research into the inhibition of EZH2 catalytic activity by small molecules is currently underway. Previously, Li et al [22] demonstrated the beneficial effects of DZNep, an EZH2 inhibitor, in an in vivo subcutaneous model of OSCC, highlighting its capability to inhibit oral cancer progression through the p16/p21 pathway and epithelial–mesenchymal transition (EMT) modulation. However, recent reports have disclosed that unlike DZNep, which is thought to deplete PRC2 subunits in cancer cells by way of an indirect mechanism [35], other EZH2 inhibitors, such as GSK343, are able to directly and selectively inhibit PRC2 enzymatic activity, probably increasing their efficacy [36]. Thus, based on these assumptions, in this study we investigated the ability of GSK343, a selective EZH2 inhibitor, to modulate the Wnt/β-catenin signaling pathway in oral cancer using an in vitro and in vivo model.

Firstly, we evaluated the cytotoxic effect of GSK343 at 24 h and 48 h in three different OSCC cell lines, CAL27, HSC-2, and HSC-3, demonstrating that GSK343 treatment significantly reduced OSCC cell viability in a concentration-dependent manner.

EZH2 has been shown to be involved in cancer cells invasion and migration, important hallmarks of malignant tumors [6,37,38]. Accordingly, GSK343 treatment, through EZH2 inhibition and H3K27 trimethylation reduction, demonstrated to significantly reduce the number of migrating cancer cells compared to those in the control group, in a concentration-dependent manner. 

The NF-κB signaling pathway is well-known to be involved in OSCC [39]. It has been demonstrated that EZH2 interacts with the NF-κB transcription factors, RelA and RelB, to activate pro-inflammatory genes in several cancer types, suggesting that the NF-κB target gene signature is positively regulated by EZH2 [40]. Following this, we discovered an increase in NF-κB expression and a decrease in IκB-α expression in the control group; however, GSK343 treatment at higher concentrations was able to significantly decrease NF-κB/IκBα pathway activation, as well as NIK expression, which is involved in the non-canonical NF-κB pathway. In the context of OSCC, some reports have demonstrated that several cytokines, particularly TNF-α, promote tumor progression by upregulating the genes associated with neutrophil recruitment and invasion, as well as IL-10, which has been associated with a more aggressive OSCC phenotype [16,17]. Following this, we found that the levels of TNF-α and IL-10, a well-known biomarker for oral cancer [41,42,43], were significantly augmented in the control group; however, GSK343 treatment modulated these inflammatory players’ release in a concentration-dependent manner, counteracting inflammation.

Recently, numerous studies have been performed that describe the significance of EZH2 in endothelial function [44,45]. As has been abundantly explained, nitric oxide (NO) is both a central regulator of endothelial function and of the innate immune response [45]. In the vasculature, NO, which is responsible for vasodilation, is produced at low concentrations by eNOS [45]; whereas, for antimicrobial defense, NO is produced in high concentrations by the inducible NO synthase (iNOS), which is regulated by cytokine-dependent transcriptional activation [45]. It has been demonstrated that iNOS can stimulate tumor angiogenesis via its product NO, which consequently increases endothelial cell migration and new capillary network formation [46,47,48]. In the latest studies, NOS2, the gene encoding iNOS, and NOS3, the gene encoding eNOS, have been identified as the target genes of EZH2 [44,45], suggesting the possible role of EZH2 in iNOS and eNOS modulation. However, despite not much being known about the crosstalk between EZH2 and the NO pathway in oral cancer, in our study we identified an increase in NO_x_, iNOS, and eNOS levels in the control group; however, treatment with GSK343 significantly reduced their levels, suggesting that EZH2 may control NO release by iNOS and eNOS modulation.

Additionally, it has been shown that EZH2 may regulate the release of several pro-angiogenic growth factors, including VEGF and TGFβ [28,49,50]. In particular, reports have shown that TGFβ, a pleiotropic factor involved in vasculogenesis and angiogenesis, promotes abnormal epithelial cell proliferation at a later stage of oncogenesis [51]. Lu and colleagues [49] revealed that TGFβ overexpression induces the hyperproliferation of cancer cells in the head and neck epithelium, suggesting that TGFβ is able to promote cell proliferation by the formation of an extracellular microenvironment that favors tumor formation [49]. Accordingly, we found an increase in VEGF expression and TGFβ levels in the control group; nonetheless, GSK343 treatment was able to significantly decrease them, demonstrating its anti-angiogenic properties.

Furthermore, oral cancer is characterized by apoptotic process dysregulation [26,52]. The process of programmed cell death known as apoptosis is necessary for normal tissue development and cell homeostasis [26]. Previous reports have demonstrated that the reduction in the proliferation and migration of OSCC cells with high EZH2 levels may be associated with an increase in apoptosis, especially via p16 and p21 regulation [6,22]. The apoptosis process has also been shown to be influenced by the NF-κB pathway, as it upregulates anti-apoptotic genes, consequently promoting cancer progression [53]. Despite various studies that have proven that EZH2 can suppress apoptosis in cancer [6,54], the exact mechanisms of apoptosis suppression by EZH2 still remain unknown. Therefore, we investigated the ability of GSK343 to modulate programmed cell death, which showed that GSK343 treatment increased the levels of pro-apoptotic proteins p53, Bax, caspase-3, and caspase-9, as well as Annexin V, suggesting that EZH2 is able to modulate the apoptosis pathway.

The effect of GSK343 on oral cancer progression was also evaluated here in an in vivo orthotopic model. Treatment with GSK343 at higher doses significantly decreased the presence of large and prominent cell nuclei, and dysplasia in tongue tissue compared to the OSCC group, reestablishing tissue architecture and counteracting tumor progression through EZH2 inhibition.

Recently, Milan and colleagues [11] revealed the involvement of EZH2 in Wnt/β-catenin signaling pathway activation, which has been shown to be associated with oral cancer progression and chemoresistance. Thus, considering these assumptions, we decided to study, in depth, the mechanism of action of GSK343 in OSCC, focusing on the Wnt/β-catenin signaling pathway. Accordingly, we discovered higher levels of β-catenin and Wnt-1 in the OSCC group compared to the sham group; however, a significant decrease in their levels was been demonstrated following GSK343 treatment, confirming the direct involvement of EZH2 in Wnt/β-catenin pathway activation in oral cancer.

In addition to EZH2, STAT3 has also been found to be hyperactive in numerous cancers [55]. EZH2 may bind to and methylate STAT3, which leads to enhanced STAT3 activity by increased tyrosine STAT3 phosphorylation, suggesting its key role in cancer [56]. In effect, our data reveal an increase in p-STAT3-positive cells in the OSCC group; nonetheless, treatment with GSK343 was able to decrease them, counteracting its activity.

Concerning the inflammatory pathway, GSK343 significantly decreased NF-κB/IκB-α pathway activation by reducing NIK expression as well, confirming the earlier in vitro data.

As previously discussed, EZH2 is a key regulator of tumor angiogenesis, as it is able to modulate the release of several angiogenic factors, including VEGF, eNOS, and TGFβ, through direct or indirect mechanisms [28,44,50,57]. Based on these considerations, our data confirm the key role of EZH2 in angiogenesis, as the OSCC group was characterized by an increase in eNOS and TGFβ expression; however, GSK343 treatment demonstrated to reduce their expression through EZH2 inhibition, proving its anti-angiogenic properties.

Furthermore, several studies have shown the usefulness of micro vessel density as a prognostic tool for patients with cancer [58,59]. CD31, a protein expressed on the surface of endothelial cells, is widely used as a prognostic marker of vascular density in malignant tissue, including oral cancer [60] as well as CD34, an important cell adhesion factor [59]. In this context, we demonstrated that GSK343 treatment was able to significantly reduce CD31 and CD34 expression, markers of micro vessel density, in OSCC.

## 4. Materials and Methods

### 4.1. In Vitro Studies

#### 4.1.1. Cell Culture

The OSCC cells (CAL27, HSC-2, and HSC-3) were obtained from American Type Culture Collection (ATCC, Rockville, MD, USA), while the oral keratinocytes (HOK) cell line was obtained from the Chinese Academy of Sciences (Shanghai, China). The CAL27 and HOK cells were grown in Dulbecco’s Modified Eagle Medium (DMEM) (Life Technologies, Gibco^®^; Carlsbad, CA, USA) with 10% fetal bovine serum (FBS, Life Technologies, Gibco^®^; Carlsbad, CA, USA), 100 U/mL penicillin, and 100 μg/mL streptomycin. The HSC-2 and HSC-3 cells were cultured in Minimum Essential Eagle’s Medium (Sigma-Aldrich; St. Louis, MO, USA) with 10% fetal bovine serum (FBS), 100 U/mL penicillin, and 100 μg/mL streptomycin (Sigma-Aldrich, St. Louis, MO, USA) at 37 °C and 5% CO_2_. The HOK cells were used as the control to evaluate the cytotoxicity of GSK343 in non-cancerous cells.

#### 4.1.2. MTT Assay 

A colorimetric assay for live cells (tetrazolium dye; MTT; Cat. no. M5655) was used to assess cell viability. After seeding the OSCC and HOK cells into 96-well plates (4 × 10^4^ cells/well), GSK343 (Cat. N°: SML0766; Sigma-Aldrich; St. Louis, MO, USA), at concentrations of 1 μM, 10 μM, 25 μM, and 50 μM, and dissolved in culture medium with 0.001% dimethyl sulfoxide (DMSO; Sigma-Aldrich; St. Louis, MO, USA), was added for 24 h and 48 h to the OSCC and HOK cells to establish the effective concentration with high toxic effects. After 24 h and 48 h of treatment, the MTT solution (0.2 mg/mL) was added for 60 min at 37 °C. The absorbance was measured at 570 nm [61]. The data from the MTT assay at 24 h were analyzed in order to calculate the LC50 (lethal concentration 50%). The values of LC50 were obtained from a probit analysis by using a regression linear curve [62].

#### 4.1.3. Cell Migration Assay

A cell migration (or scratch) assay was performed after 24 h of treatment, as previously described in [63]. Images of the wound were recorded immediately after wounding (0 h) and after culturing (24 h), using an inverted research microscope. Image J software, version 1.46, was used to evaluate the number of migrated cells into the scratched area.

#### 4.1.4. Immunoblotting

Immunoblotting was executed after 24 h of treatment to measure the expression of anti-nuclear factor of kappa-light-chain-enhancer in B cells (NF-κB) (1:500; Santa Cruz Biotechnology, Dallas, TX, USA; sc-8008), inhibitor nuclear factor of kappa-light-chain-enhancer in B cells α (IκBα) (1:500; Santa Cruz Biotechnology, Dallas, TX, USA; sc-1643), vascular endothelial growth factor (VEGF) (1:500; Santa Cruz Biotechnology, Dallas, TX, USA; sc-7269), and endothelial nitric oxide synthase (eNOS) (1:500; Santa Cruz Biotechnology, Dallas, TX, USA; sc-376751) in the CAL27 cells [26]. Glyceraldehyde-3-Phosphate Dehydrogenase (GAPDH) (1:1000; Santa Cruz Biotechnology; Dallas, TX, USA, sc-365062) was used as the loading control.

#### 4.1.5. NO_x_ Assay

After 24 h of treatment, a Griess assay was used to measure the total NO_x_ levels in the CAL27 supernatants, as previously reported in [64].

#### 4.1.6. Enzyme-Linked Immunosorbent Assay (ELISA) for EZH2, H3K27me3, NIK, IKKβ, TNFα, IL-10, TGFβ, iNOS, and Annexin V

ELISA kits were used to quantify the levels of EZH2, NIK, IKKβ, TNF-α, IL-10, TGFβ, iNOS, and Annexin V in the CAL27 supernatants (Human EZH2 Elisa kit My BioSource, cat. No. MBS2608111; Human NIK ELISA kit Biobool, cat. No. E024745; Human IKKβ ELISA kit RayBiotech, cat. No. ELH-IKKB-1; Human TNF-α ELISA Kit My BioSource, cat. No. MBS267654; Human IL-10 ELISA Kit My BioSource, cat. No. MBS2700942; Human TGFβ ELISA kit RayBiotech, cat. No. ELH-TGFb1; Human iNOS ELISA kit BT Lab, cat. No. E4710Hu; Human Annexin V SimpleStep ELISA Kit Abcam, cat. No. ab223863) in accordance with the standard procedure described in each protocol. Moreover, after histone extraction, we measured the percentage of H3K27 trimethylation (Human Histone H3 (tri-methyl K27) Assay Kit, Abcam, cat. No. ab115051) in the CAL27 cells according to the protocol.

#### 4.1.7. RNA Isolation, cDNA Synthesis, and Real-Time Quantitative PCR Amplification

A Trizol Reagent Kit (Life Technologies, Monza, Italy) was used to obtain the total RNA from the CAL27 cells [52] to assess p53, Bax, caspase-3, and caspase-9 gene expression after 24 h of treatment. β-actin was used for the normalization. The data were expressed as a fold change. Table 1 illustrates the primer used for the RT-qPCR.

### 4.2. In Vivo Studies

#### 4.2.1. Cell Line 

The CAL27 cells were obtained from ATCC (Rockville, MD, USA) and were grown as described in Section 4.1.1.

#### 4.2.2. Animals

BALB/c nude mice (male, 8–10 weeks old) were obtained from Jackson Laboratory (Bar Harbor, Hancock, ME, USA) and housed in accordance with ARRIVE procedures in microisolator cages under pathogen-free conditions on a 12 h light/12 h dark schedule for a week. The animals were fed a standard diet and allowed water ad libitum. The animal experiments respected Italian regulations on the protection of animals (DM 116192) and EU regulations (European Directive 2010/63/EU, amended by Regulation 2019/1010). The University of Messina approved this animal study (n◦ 368/2019-PR, released on 14 May 2019).

#### 4.2.3. OSCC Orthotopic Model

CAL27 orthotopic model was designed as previously mentioned in [65,66]. Briefly, one injection of 5 × 10^6^ CAL27 cells in 25 μL of phosphate-buffered saline (PBS) (Sigma-Aldrich; St. Louis, MO, USA) was performed into the lateral portion of the tongue of the animals using a sterile 0.5 mL insulin syringe with a 30-gauge needle [65]. The mice in the control group received only the vehicle. After 21 days, the mice were randomly separated into 4 groups to receive either the vehicle or GSK343 (1 mg/kg, 5 mg/kg, and 10 mg/kg) every 3 days by intraperitoneal injection. During the experiment, serum collection was performed every week. The mice were checked each day for signs of morbidity for the survival study.

At the end of the experiment, the animals were euthanized as previously described in [67]; the tongue and serum samples were collected to perform several analyses.

The statistical test “ANOVA: Fixed effect, omnibus one-way” with G-power software was used to assess the minimum number of mice for each technique, generating a sample size equal to *n* = 10.

The GSK343 doses were selected in accordance with a dose–response study executed in our laboratory.

The sham group + GSK343 10 mg/kg experimental data were not included because they did not present any differences in improvement or toxicity when compared to the sham group.

Furthermore, the OSCC group + GSK343 1 mg/kg was only examined for body weight, histology, and EZH2 ELISA kit because it did not show any positive results.

#### 4.2.4. Histological Evaluation

A histological assessment was performed as previously explained [26]. A pathologist blinded to the treatment groups examined the slides using a microscope Axiovision (Zeiss, Milan, Italy). The parameters considered for the analysis were (i) the normal epithelium, (ii) hyperplasia, (iii) mild dysplasia, (iv) moderate dysplasia, (v) severe dysplasia, and (vi) carcinoma [68].

#### 4.2.5. Immunohistochemistry for β-Catenin and p-STAT3

Paraffin-embedded tongue samples were segmented at 7 μm, deparaffinized, and processed as previously reported [26]. The sections were incubated overnight at 4 °C with β-catenin antibody (1:100, Santa Cruz Biotechnology, Dallas, TX, USA; sc-133238) and p-STAT3 (1:100, Santa Cruz Biotechnology, Dallas, TX, USA; sc-8059). The sections were examined using a Nikon Eclipse Ci-L microscope.

#### 4.2.6. Enzyme-Linked Immunosorbent Assay (ELISA) for EZH2, H3K27me3, Wnt-1, β-Catenin, and STAT3

ELISA kits were used to quantify the levels of EZH2, β-catenin, Wnt-1, and p-STAT3/total STAT3 (Mouse EZH2 ELISA kit, Catalogue No: abx522587, Abbexa; Mouse beta Catenin ELISA kit, Catalog Number: A74741, Antibodies-online.com; Mouse Wnt-1 ELISA kit, Catalog Number: MBS2883286, My BioSource; Mouse p-STAT3/total STAT3 ELISA kit, Catalogue Number: ABIN625245, Antibodies-online.com) in the tongue samples in accordance with each protocol. Moreover, after histone extraction, we measured the levels of H3K27me3 (EpiQuik^TM^ Circulating Trimethyl Histone H3K27 ELISA Kit, Catalog Number: P-3124) in the serum samples.

#### 4.2.7. Immunoblotting

Immunoblotting was executed to measure the expression of NF-κB (1:500; Santa Cruz Biotechnology, Dallas, TX, USA; sc-8008), IκBα (1:500; Santa Cruz Biotechnology, Dallas, TX, USA; sc-1643), NIK (1:500; Santa Cruz Biotechnology, Dallas, TX, USA; sc-6363), eNOS (1:500; Santa Cruz Biotechnology, Dallas, TX, USA; sc-376751), TGFβ (1:500, Santa Cruz Biotechnology, Dallas, TX, USA; sc-130348), CD34 (1:500; Santa Cruz Biotechnology, Dallas, TX, USA; sc-74499), and CD31 (1:500, Santa Cruz Biotechnology, Dallas, TX, USA; sc-376764) in the cytosolic and nuclear extracts of the tongue tissues [69]. Lamin A (1:1000; Santa Cruz Biotechnology; Dallas, TX, USA; sc-518013) and GAPDH (1:1000; Santa Cruz Biotechnology; Dallas, TX, USA; sc-365062) were used as the loading controls for the nuclear and cytosolic fraction, respectively.

### 4.3. Materials

GSK343 (Cat. N°: SML0766) and additional substances were obtained from Sigma-Aldrich (Milan, Italy). DMSO and PBS were used to prepare the stock solutions (Sigma-Aldrich).

### 4.4. Statistical Analysis 

The in vitro and in vivo results are expressed as the mean ± standard deviation (SD) of “n” observations. A one-way ANOVA followed by Bonferroni’s multiple comparison test and a two-way ANOVA were used to examine the data. A *p*-value of less than 0.05 was considered significant. GraphPad Prism 7.04 software was used for all the statistical analyses.

## 5. Conclusions 

In summary, all this information offers a new perspective about the role of EZH2 in OSCC pathogenesis in comparison to previous studies, especially concerning its function in NO pathway modulation. Moreover, based on our results, GSK343, a selective EZH2 inhibitor, could represent a promising pharmacological strategy for OSCC management by modulating the Wnt/β-catenin signaling pathway and the expression of important inflammatory markers associated with OSCC progression. However, taking into consideration the lack of additional data with knock-out (KO) cells or other inhibitors, and the limitations of preclinical models in the translational reproduction of human diseases, further studies are required to better investigate the efficacy and molecular mechanisms of GSK343 in OSCC.

## Figures and Tables

**Table 1:** Primer sequences.

Gene	Forward Primer	Reverse Primer
p53	-AGAGTCTATAGGCCCACCCC-	-GCTCGACGCTAGGATCTGAC-
Bax	-GGACGAACTGGACAGTAACATG-	-GCAAAGTAGAAAAGGGCGACA-
Caspase-3	-CTGAGGCATGGTGAAGAAGGA-	-GTCCAGTTCTGTACCACGGCA-
Caspase-9	-TGCGAACTAACAGGCAAGCA-	-GTCTGAACCTCTCTGGTTTGC-
β-actin	-GACTTCGAGCAAGAGATGG-	-AGCACTGTGTGGCGTACAG-

## Data Availability

All data generated or analyzed during this study are included in this article.

## Supplementary Materials

The following supporting information can be downloaded at: https://www.mdpi.com/article/10.3390/ph17081102/s1, Figure S1: Effect of GSK343 on HOK cell viability at 24 h and 48 h.

## Abbreviations

Head and neck squamous cell carcinoma	(HNSCC)
Oral squamous cell carcinoma	(OSCC)
Enhancer of zeste homolog 2	(EZH2)
Histone H3 lysine27	(H3K27)
Polycomb 2 repressive complex	(PRC2)
GlaxoSmithKline 343	(GSK343)
Wnt family member 1	(Wnt-1)
Signal transducer and activator of transcription 3	(STAT3)
Nuclear factor κ-light-chain-enhancer of activated B cells	(NF-κB)
Inhibitor nuclear factor of kappa-light-chain-enhancer in B cells α	(IκBα)
Tumor necrosis factor-α	(TNF-α)
Interleukin-10	(IL-10)
Vascular endothelial growth factor	(VEGF)
Endothelial nitric oxide synthase	(eNOS)
Transforming growth factor β	(TGFβ)
