# Supplementary material for: EZH2 Inhibition to Counteract Oral Cancer Progression through Wnt/β-Catenin Pathway Modulation
Source: Pharmaceuticals (Basel). 2024 Aug 22;17(8):1102. doi: 10.3390/ph17081102 (PMC11357505; doi:10.3390/ph17081102)
Supplement: Supplementary file 1 [file pharmaceuticals-17-01102-s001.zip › pharmaceuticals-3156291-supplementary.pdf]

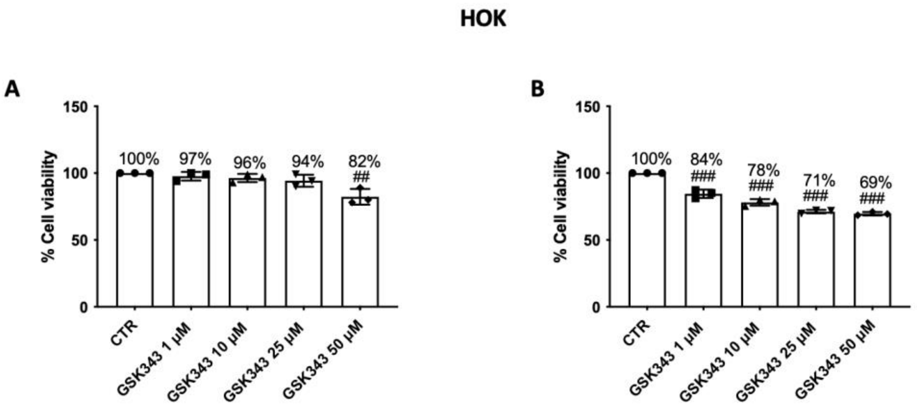

Supplementary Figure 1. Effect of GSK343 on HOK cell viability at 24 h and 48 h. GSK343 did not exert any cytotoxic effect on non-cancerous HOK cells after 24 h of treatment, however the highest concentration reduced HOK cell viability to 82% (A). After 48 h of treatment, GSK343 (1, 10, 25 and 50 μM) significantly reduced HOK cell viability by up to 69% compared to the control group (B). The data were obtained from three independent experiments. (A) ## $p < 0.01$ vs. control group. (B) ### $p < 0.001$ vs. control group.
